# A Long-Term Prediction Model of Beijing Haze Episodes Using Time Series Analysis

**DOI:** 10.1155/2016/6459873

**Published:** 2016-08-14

**Authors:** Xiaoping Yang, Zhongxia Zhang, Zhongqiu Zhang, Liren Sun, Cui Xu, Li Yu

**Affiliations:** ^1^School of Information, Renmin University of China, Beijing 100872, China; ^2^School of Computer Science, Northeastern University, Shenyang 110819, China

## Abstract

The rapid industrial development has led to the intermittent outbreak of pm2.5 or haze in developing countries, which has brought about great environmental issues, especially in big cities such as Beijing and New Delhi. We investigated the factors and mechanisms of haze change and present a long-term prediction model of Beijing haze episodes using time series analysis. We construct a dynamic structural measurement model of daily haze increment and reduce the model to a vector autoregressive model. Typical case studies on 886 continuous days indicate that our model performs very well on next day's Air Quality Index (AQI) prediction, and in severely polluted cases (AQI ≥ 300) the accuracy rate of AQI prediction even reaches up to 87.8%. The experiment of one-week prediction shows that our model has excellent sensitivity when a sudden haze burst or dissipation happens, which results in good long-term stability on the accuracy of the next 3–7 days' AQI prediction.

## 1. Introduction

Industry of developing countries is mainly centralized around big cities, accompanied by a large population, consumption, and pollution. Together with Tianjin city and Hebei province, Northern China has become one of the most prosperous and polluted areas on Earth. By 2013, the transient population of Beijing was 37.5 million, and the intermittent outbreak of air pollution has greatly impacted every citizen's life: physiological diseases [1, 2], depression, and poor visibility in traffic [3, 4]. The main component of haze is pm2.5 (particulate matters less than 2.5 *μ*m in aerodynamic diameter), and the concentration of pollution is described with Air Quality Index (AQI, the concentration of pm2.5). The Chinese Government began to monitor and record pm2.5 concentrations for major cities since 2013 [5]. According to the report of Quan et al. [6], the AQI reached 600 in Beijing during the haze event in January 2013. In recent years, more and more papers have referred to the haze episodes and the consequences in Northern China [7–11]. Researchers pointed out that, over the coming years, haze episodes would continue to burst frequently in Northern China [12].

This paper presents an AQI prediction model of Beijing based on time series analysis. We collected Beijing's AQI data of 29 continuous months since 2013 and constructed a dynamic structural prediction model. Statistical methods are used to obtain the maximum likelihood estimation of the prediction model. And both short-term and long-term experiments are carried out to test the accuracy and robustness of our model.

The remainder of this paper is organized as follows. In Section 2, we introduce recent related work.  Section 3 presents our prediction model and proves our model to be a vector autoregressive model. Experiments and evaluations are reported in Section 4. We conclude the paper in Section 5 with future works.

## 2. Related Work

Generally, pm2.5, or haze, is born mainly through anthropogenic factors [13–16] and eliminated by natural diffusion. Several days after emission, secondary pm2.5 is produced through photochemical reactions among indiffusible pollutants. Secondary pm2.5 is the principal component in most severe haze episodes in China [17]. A typical way of haze prediction is to use pollutant emission data (CO, SO_2_, and NO_*x*_) in the simulation [5, 18]. Huang et al. [14] analyzed the chemical compositions of pm2.5 and used chemical mass balance to identify the emission sources. Other more complex models are proposed to introduce the atmospheric features, chemistry components, and transport factors [15]. But the more common case is that pollutant emission data usually increase or decrease synchronously with AQI. Sun [19] took population, car ownership, and GDP into consideration and proposed a statistical index system of average annual haze episode days. They found that although most factors contribute to predicting pm2.5, the annual average of NO_*x*_ is negatively correlated with average severely polluted days. The paper [12] established a cubic exponential smoothing model by introducing dust emission into haze prediction. Liang et al. pointed out that there are various distribution and transmission patterns of pm2.5 [20]. In fact, Wang et al. mentioned that the government control policy should be considered in model simulations [9].

Many researches use backpropagation neural network as the simulation model [19, 21]. Statistical time series analysis is rarely used in haze prediction, so long-term haze prediction is difficult for current methods to accomplish [22]. Multiple linear regression models also perform well on daily scale prediction [23, 24]. However, the test data of existing researches is not ample; for example, [21] tested the prediction accuracy on only 3 days. Besides, Zhang et al. pointed out that pm2.5 accumulation in previous days significantly affects the present daily pm2.5 concentration, which should also be a concern in the modeling process [22].

Considering the above points, this paper presents a new AQI prediction model integrated with natural factor, humanity factor, and self-evolution factor.

## 3. The Prediction Model of Beijing's Daily AQI

### 3.1. The Parameters and Architecture of the Prediction Model

The change of daily pm2.5 concentration depends on two factors: daily overall production of pm2.5 by human activities *P*_*t*_ and daily overall natural diffusion or overall natural accumulation of pm2.5 *C*_*t*_. The production of haze depends a lot on the control policies of the government toward the emission of industry fuels *I*_*t*_. The diffusion of haze mainly depends on the airflow *W*_*t*_. Besides, complex chemical changes could occur between pm2.5 and other pollutants; thus, previous day's pm2.5 concentration also affects the AQI, which could be seen as the evolution result of previous day's pm2.5 and is represented by *Y*_*t*_. Apparently, *P*_*t*_ − *C*_*t*_ could be directly observed. *P*_*t*_ is generated by a semimanual method. *P*_*t*_ is mainly related to daily human activities, and we calculate *P*_*t*_ from AQI sequences of no less than five consecutive sunny and windless days. Special circumstances are also considered. In winter, *P*_*t*_ will be larger because the heating system is on. The car usage restrictions and temporary stoppage of factories during Beijing APEC 2014 are also taken into consideration. *C*_*t*_ is then calculated as *P*_*t*_ − (*P*_*t*_ − *C*_*t*_). Sometimes, *C*_*t*_ is greater than zero, which means pm2.5 accumulates because of nonhuman factors.

Thus, the daily net growth of pm2.5 (*P*_*t*_ − *C*_*t*_) is a function of the evolution result *Y*_*t*_, the industry control index *I*_*t*_, and the forecast of wind power *W*_*t*_. Consider this problem as a dynamic structural model, and our model can be described as

(1) Pt−Ct=β0+β1Yt+β2Wt+β3It+β4Pt−1−Ct−1+μtD.

Parameters *β*_1_, *β*_2_, and *β*_3_, respectively, represent the effect caused by the pm2.5 of the previous day, the wind power, and the industry control index. The net growth of previous day's pm2.5 partly affects present day's pm2.5 and partly affects the next day's pm2.5. The parameter *β*_4_ represents this “partial adjustment.” The disturbance *μ*_*t*_^*D*^ represents other factors which affect present day's pm2.5.

### 3.2. Complexity Reduction of the Prediction Model

In order to facilitate the research and modeling process, we have proved that this model could be reduced to a vector autoregressive model.

**Proposition 1 .**

Formula (1) is a vector autoregressive model.

**Proof**

Assume that there exists sequence autocorrelation in formula (1). The autocorrelation is

(2) $$\begin{array}{c} \mu_{t}^{D}=\rho \mu_{t-1}^{D}+\upsilon_{t}^{D} \end{array}$$

in which *υ*_*t*_^*D*^ is white noise. Here, we apply the Cochrane-Orcutt iteration to rewrite formula (2):

(3) $$\begin{array}{c} \left(\;1-\rho L\;\right)\mu_{t}^{D}=\upsilon_{t}^{D}, \end{array}$$

where *L* is the lag operator (*LV*_*t*_ ≡ *V*_*t*−1_), which can convert the last phase to current value in a time series.

The next work is to find the most satisfying value of *ρ* through successive iteration method. Specifically, this method uses residual error to estimate the unknown *ρ*.

Assume that we use previous *p* days' AQI to predict present day's AQI. Multiply (1 − *ρL*) on both sides of formula (1); the expansion formula will be as follows:

(4) Pt=k1+β120Ct+β130It+β140Yt+β150Wt+β111Pt−1+β121Ct−1+β131It−1+β141Yt−1+β151Wt−1+β112Pt−2+β122Ct−2+β132It−2+β142Yt−2+β152Wt−2+⋯+β11pPt−p+β11pCt−p+β11pIt−p+β11pYt−p+β11pWt−p+υtD.

In the substitution process, many assumptions are neglected. But the ordinary least square method (OLS estimation) should not be used in the estimation of formula (4), because OLS can only illustrate the relationship between daily pm2.5 production and the policy control index, the accumulation of history pm2.5, and the wind power. The net growth of previous day's pm2.5 is only one reason of the correlation of these variables.

The government could make policies to control pm2.5 production of industry to obtain “satisfying” daily production of pm2.5; that is, *I*_*t*_ is an endogenous variable. And the policy control index depends on present day's and previous *p* days' accumulation of history pm2.5, the wind power, the daily production of pm2.5, and daily diffusion of pm2.5:

(5) It=k3+β310Pt+β320Ct+β330Yt+β340Wt+β311Pt−1+β321Ct−1+β331Yt−1+β341Wt−1+β351It−1+β312Pt−2+β322Ct−2+β332Yt−2+β342Wt−2+β352It−2+⋯+β31pPt−p+β32PCt−p+β33PYt−p+β34PWt−p+β35PIt−p+υtC,

where *υ*_*t*_^*C*^ represents the influence brought about by other policies.

The net growths of previous days' pm2.5 and policy control index also have an effect on daily accumulation of pm2.5:

(6) Yt=k4+β410Pt+β420Ct+β430It+β440Wt+β411Pt−1+β421Ct−1+β431It−1+β441Wt−1+β451Yt−1+β412Pt−2+β422Ct−2+β432It−2+β442Wt−2+β452Yt−2+⋯+β41pPt−p+β42pCt−p+β43pIt−p+β44pWt−p+β45pYt−p+υtA,

where *υ*_*t*_^*A*^ represents other factors that influence daily accumulation of pm2.5.

Analogized from formulas (4), (5), and (6), *C*_*t*_ and *W*_*t*_ can both be written in a similar form. Join formulas (4), (5), and (6) together, and rewrite them into vector form:

(7) $$\begin{array}{c} B_{0}x_{t}=K+B_{1}x_{t-1}+B_{2}x_{t-2}+\cdots +B_{p}x_{t-p}+\upsilon_{t} \end{array}$$

in which

(8) xt=Pt,Ct,It,Yt,WtT,υt=υtD,υtS,υtC,υtA,υtHT,K=k1,k2,k3,k4,k5,B0=1−β120−β130−β140−β150−β2101−β230−β240−β250−β310−β3201−β340−β350−β410−β420−β4301−β450−β510−β520−β530−β5401.

In *B*_0_, the parameters in the 1st, 2nd, 3rd, 4th, and 5th row, respectively, relate *P*_*t*_, *C*_*t*_, *I*_*t*_, *Y*_*t*_, and *W*_*t*_ to the other variables. Every *B*_*s*_ is a 5*∗*5 matrix. Premultiply formula (7) by *B*_0_^−1^ (the inverse matrix of *B*_0_):

(9) $$\begin{array}{c} x_{t}=c+\Psi_{1}x_{t-1}+\Psi_{2}x_{t-2}+\cdots +\Psi_{p}x_{t-p}+\varepsilon_{t} \end{array}$$

in which

(10) c=B0−1K,Ψs=B0−1Bs,εt=B0−1υt.

This is the standard form of vector autoregressive model. So it is proved that our prediction model (formula (1)) is in fact a vector autoregressive model.

The regression parameters of our haze prediction model can be obtained as follows.

Let

(11) −Γ=KB1B2⋯Bp,yt=1xt−1xt−2⋯xt−pT.

The dynamic structural system (formula (7)) isss

(12) $$\begin{array}{c} B_{0}x_{t}=-\Gamma x_{t}+\upsilon_{t}. \end{array}$$

Assume that the disturbance terms are not sequence correlated or correlated to each other, which means

(13) $$\begin{array}{c} E\left(\;\upsilon_{t}\upsilon_{\tau }^{T}\;\right)=\left\{\begin{array}{cc} D, & t=\tau ,\\ 0, & t\neq \tau . \end{array}\right. \end{array}$$

*D* is a main diagonal matrix. Formula (12) could be written as

(14) $$\begin{array}{c} x_{t}=\Phi^{T}y_{t}+\varepsilon_{t} \end{array}$$

in which

(15) ΦT=B0−1−Γ,εt=B0−1υt.

Let *Ω* be the variance-covariance matrix of *ε*_*t*_:

(16) $$\begin{array}{c} \Omega =E\left(\;\varepsilon_{t}\varepsilon_{t}^{T}\;\right)=B_{0}^{-1}E\left(\;\upsilon_{t}\upsilon_{t}^{T}\;\right){\left(\;B_{0}^{-1}\;\right)}^{T}=B_{0}^{-1}D{\left(\;B_{0}^{-1}\;\right)}^{T}. \end{array}$$

Suppose *B*_0_ is a lower triangular matrix, in which all main diagonal elements are assigned 1, and *D* is a main diagonal matrix. The parameters (*B*_0_, Γ, *D*) can be obtained through the maximum likelihood estimation of complete information. The maximum likelihood estimation of *Ω* can be obtained by the variance-covariance matrix of regression residual.

Finally, ${\overset{⌢}{B}}_{0}^{-1}$ and *D* are calculated through triangular decomposition of $\overset{⌢}{\Omega }$; thus, Γ can be evaluated.

Above all, the prediction model of Beijing AQI has considered factors including industry emission and policy control, together with the chemical changes of previous days' pollution accumulation and the diffusion conditions. This model also takes the correlations between these factors into consideration and introduces time series haze features into the dynamic structural model. The policy control index is simulated by the record of 4 severe haze episodes during this period. The diffusion is evaluated by weather record of daily wind power.

## 4. Model Evaluations

We collected the daily AQI and daily weather information from 28 Oct. 2013 to 31 Mar. 2016. This complete sequence is used to test the accuracy of the prediction model. The next day's AQI prediction experiment (Section 4.1) and long-term AQI prediction experiment (Section 4.2) are both implemented. The next day's AQI prediction is evaluated from two perspectives: the accuracy of daily prediction and the accuracy of statistical analysis.

### 4.1. Next Day's AQI Prediction

**Figure 1:**
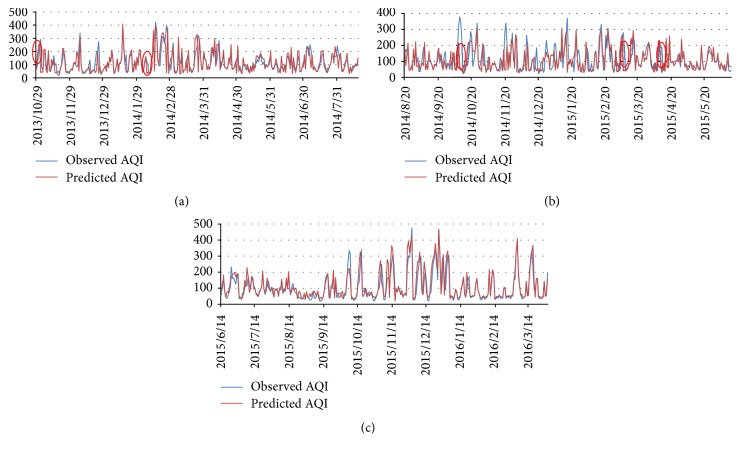
(a, b, c) Next day's AQI prediction on 886 continuous days.

The next day's weather forecast information is applied in next day's AQI prediction. The observed and predicted daily mean AQI in Beijing are illustrated in Figure 1. The simulation result shows that the predicted value matched the observed value very well on the whole sequence of 886 days. Sometimes, there is severe deviation from the observed value; for example, on 19 Feb. 2014, the observed AQI was 89, while our model gives a prediction of 209, with an offset of 135%. But the fact is, in the afternoon of 19 Feb., the wind of Beijing suddenly changed from northeasterly to southwesterly, and by 19:00 the AQI has reached already up to 170, which could be interpreted as our model successfully forecasted a severe haze outbreak several hours in advance; in the coming 7 days, the average daily AQI of Beijing is 305. The occasional occurrence of this “foreseeing” phenomenon is caused by coarse time granularity (daily), and this phenomenon is marked with red ellipse in Figure 1. These marks indicate that our model could “foresee” the sharp change of both outbreaks and diffusions. Most haze outbreaks and diffusions could be accurately simulated; some could be foreseen but could never be delayed.

**Figure 2:**
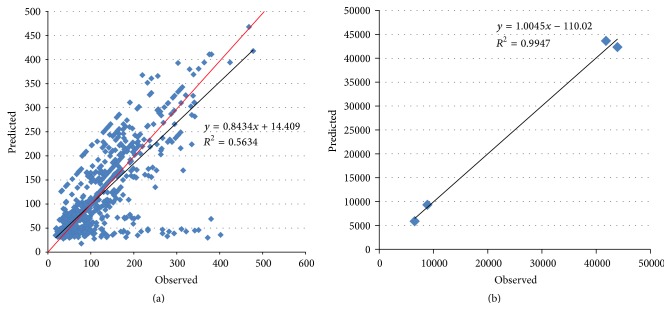
(a) Daily AQI of the 886 days. (b) Annual AQI from 2013 to 2016.

Figure 2(a) is a scatter diagram of daily AQI, including both observed value and predicted value. Most points lie close to *y* = *x* (the red line). But some points lie in a queue at the bottom part, which means the observed AQI exceeds 200, while the predicted value is less than 50. There are altogether 15 such outliers, 7 of which “foresee” haze diffusion, while the other 8 bug points could not be well interpreted. All the 15 points are checked and listed in Table 1. “✓” means a “foreseeing” phenomenon, and “?” represents bug points.  Figure 2(b) is a scatter diagram of annual AQI (sum of daily AQI in a certain year). Our data covers only 2 months of 2013 and 3 months of 2016, so, in this diagram, these 2 points lie in the lower left corner.

**Figure 3:**
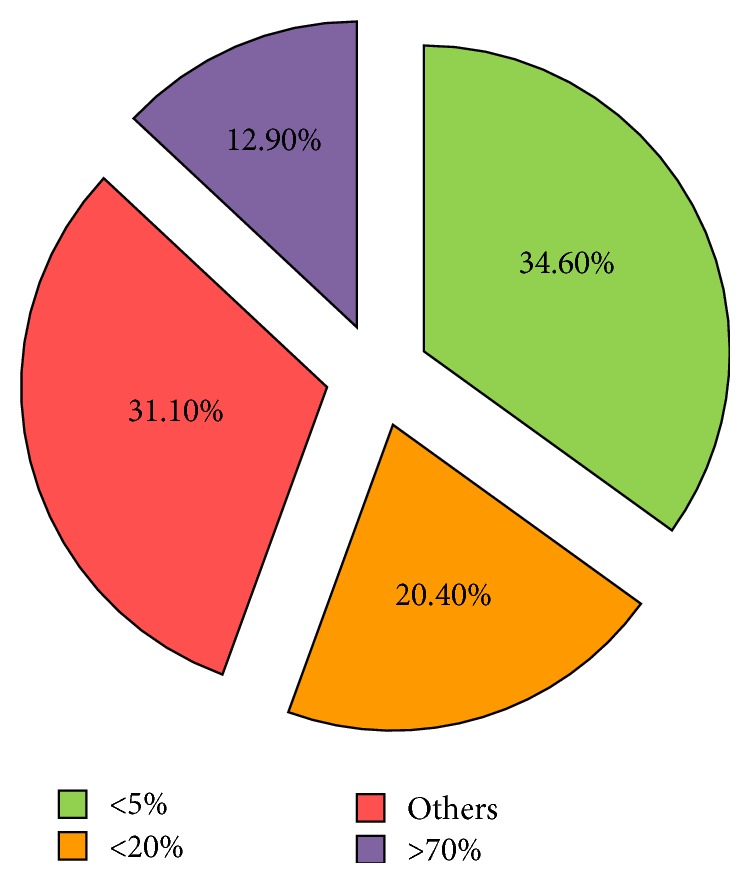
The deviation of predicted and observed AQI.

The pie chart in Figure 3 shows the distribution of prediction accuracy. The deviation of predicted and observed AQI is obtained through the following formula:

(17) $$\begin{array}{c} Dev_{t}=\frac{\left|PredictedValue-ObservedValue\right|}{ObservedValue}\times 100\%. \end{array}$$

**Figure 4:**
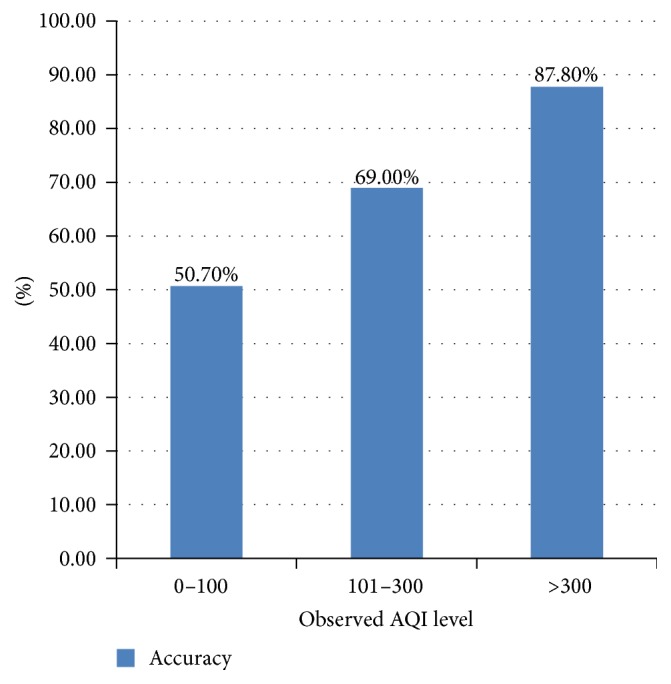
Prediction accuracies of different air qualities.

Figure 3 shows that 55% predictions match the observed values very well (<20% deviation). The purple part is mainly caused by the “foreseeing” phenomenon. Most samples of the red part come from less-polluted days. For example, on 12 Jan. 2016, the AQI prediction is 40 while the observed AQI is 29, which makes a deviation of 37.9%. In fact, statistics also indicate that our model performs better in worse air conditions (Figure 4). A sample is correctly predicted if the deviation of a sample is less than 20% or the predicted air quality level matches the observed level.

### 4.2. Long-Term AQI Prediction

**Figure 5:**
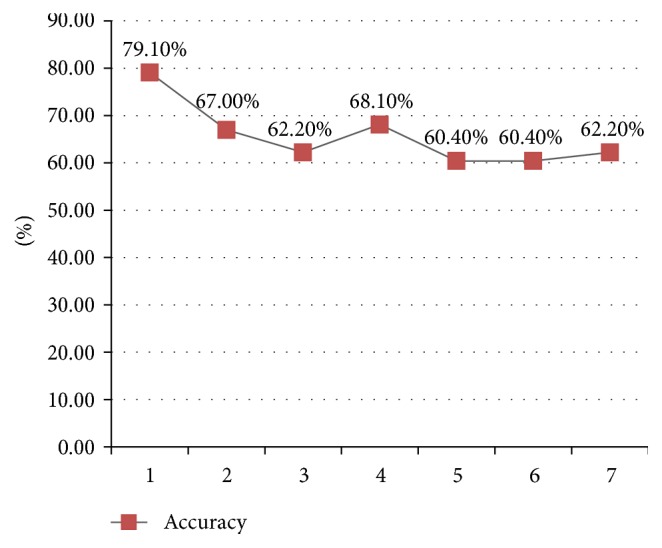
The accuracy of long-term AQI prediction.

**Figure 6:**
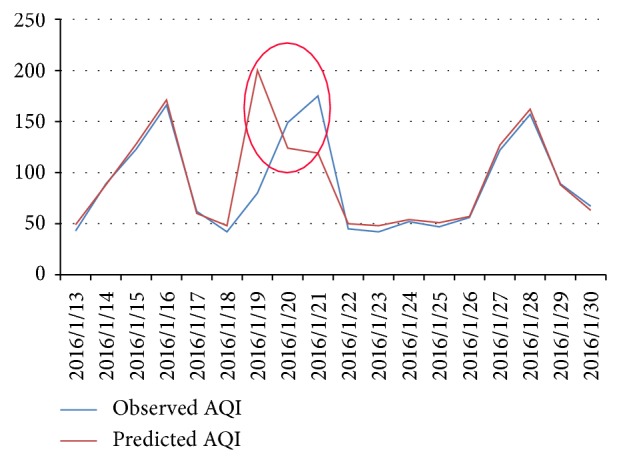
Three haze episodes in Jan. 2016.

**Figure 7:**
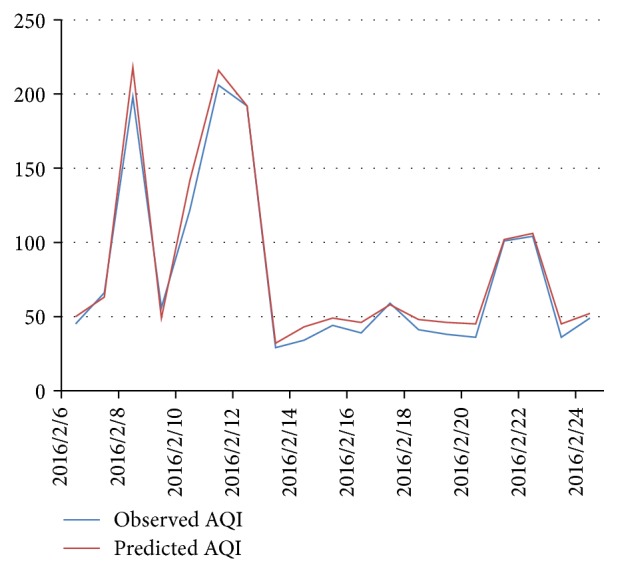
Three haze episodes in Feb. 2016.

In the long-term prediction, we use history haze data sequence and weather forecast information to predict the next 7 days' AQI. A sample is correctly predicted if the deviation of a sample is less than 20% or the predicted air quality level matches the observed level. From 26 Dec. 2015 to 31 Mar. 2016, we predict the AQI in the next 7 days and check the accuracy of *n*-day predictions.  Figure 5 shows the accuracy of long-term prediction in the 91 days' experiment.  Figure 5 shows that the accuracy stays stable on the next 3, 4, 5, 6, and 7 days' AQI prediction, which indicates that our model has excellent robustness on the task of long-term prediction. The next day's prediction accuracy surprisingly reaches 79.1%, which is far better than the experiment in Section 4.1. The reason is that, during the 91 days, 6 haze episodes attacked Beijing. These frequent attacks did contribute a lot to the overall performance because our model is very sensitive to sudden changes of AQI, including outbreaks and diffusions (Section 4.1; Figure 4). Figures 6 and 7 show several haze episodes during the 91 days. Both figures show a pm2.5 change process of more than 2 weeks.  Figure 6 also shows a “foreseeing” phenomenon caused by coarse time granularity, marked by a red ellipse.

## 5. Conclusion and Future Work

This paper presented a dynamic structural model to predict Beijing's daily AQI. This model integrated natural factor, humanity factor, and self-evolution factor into the time series model. This dynamic structural measurement model of daily haze increment is proven to be a vector autoregressive model. Experiments reflected two highlights of this model. First, our model is very sensitive to and performs very well on predicting sudden changes of AQI, including both outbreaks and diffusions. Second, the model has great robustness on the task of long-term AQI prediction. Lastly, limited by the coarse time granularity, our model sometimes “foresees” but never delays or misses any sudden changes of haze.

Many researchers use simple backpropagation neural network to accomplish nonlinear prediction models. But since methods based on time series are proven to be effective in haze prediction modeling, we believe that recurrent neural networks give better performances in such a prediction task. Although the related factors are limited in existing models, the overfitting problem should still be concerned, because, in long-term prediction, a deviation could spread and be exaggerated in the following days' predictions.

## Figures and Tables

**Table 1:** All the 15 outliers in Figure 2(a).

Date of outlier	Label
Nov. 2, 2013	*✓*
Dec. 7, 2013	?
Dec. 25, 2013	*✓*
Feb. 14, 2014	?
Feb. 25, 2014	?
Mar. 26, 2014	?
Oct. 10, 2014	*✓*
Oct. 11, 2014	*✓*
Nov. 19, 2014	?
Nov. 20, 2014	?
Nov. 30, 2014	*✓*
Dec. 9, 2014	?
Jan. 4, 2015	?
Jan. 15, 2015	*✓*
Mar. 7, 2015	*✓*
